# Microbial Desalination Using *Advenella faeciporci* ZF1: A Novel Iron-Reducing Bacterium Isolated from Oil Refinery Sludge

**DOI:** 10.21203/rs.3.rs-7905108/v1

**Published:** 2025-11-28

**Authors:** Zarrindokht Emami-Karvani, Giti Emtiazi, Iraj Nahvi, Alimohammad Ahadi, Kianoush Khosravi-Darani

**Affiliations:** 1. Department of Microbiology, Fal.C., Islamic Azad University, Isfahan, Iran.; 2. Department of Biology, Faculty of Sciences, University of Isfahan, Isfahan, Iran.; 3. Department of Genetic, Faculty of Science, University of Sharekord, Shahr-e Kord, Iran.; 4. Research Department of Food Technology, National Nutrition and food Technology Research Institute, Faculty of Food and Nutrition Sciences, Shahid Beheshti University of Medical Sciences, P.O. Box: 19395-4741, Tehran, Iran.

**Keywords:** Microbial Desalination Cells (MDCs), Iron-Reducing Bacteria (IRB), Bioelectricity generation, Electroactive Bacteria in Desalination, Microbial fuel cells (MFCs)

## Abstract

*Advenella faeciporci* strain ZF1, a novel isolated iron-reducing bacterium from Esfahan Oil Refining sludge, exhibited remarkable tolerance to FeCl_3_ (up to 20 g/L) and electrochemical activity under aerobic and anaerobic conditions. ZF1 was phylogenetically identified and characterized via FTIR, polyhydroxybutyrate (PHB) accumulation, and magnetotactic behavior. Integrated into a microbial desalination cell (MDC), ZF1 achieved sodium, calcium, and chloride removal efficiencies of 70.6%, 78.6%, and 27.5% in refinery brine over 15 days. Under 9 g/L NaCl salinity, the system generated a maximum voltage of 410 ± 15 mV within the first 48 h. The corresponding peak power density reached 92 ± 5 mW/m^2^ at an external resistance of 100 Ω, and the calculated current density was 1.80 ± 0.1 A/m^2^. These findings highlight *A. faeciporci* ZF1 as a promising exoelectrogen for sustainable water desalination and metal-rich wastewater bioremediation.

## 1. Introduction

Freshwater scarcity, driven by global population growth, industrial expansion, and climate variability, has intensified the need for sustainable desalination technologies. Conventional systems such as reverse osmosis and electrodialysis are effective but energy-intensive and often generate concentrated brine waste [1]. In contrast, microbial desalination cells (MDCs) have emerged as a promising alternative that integrates desalination, wastewater treatment, and bioelectricity generation into a single bioelectrochemical platform [2,3].

MDCs operate by harnessing the metabolic activity of electroactive bacteria—also termed exoelectrogens—which oxidize organic matter at the anode and transfer electrons to an external circuit. This creates an electric potential that drives ion migration across selective membranes: cations migrate through a cation exchange membrane (CEM) to the cathode, while anions pass through an anion exchange membrane (AEM) to the anode [4]. The efficiency of such systems is critically dependent on the metabolic robustness and electron transfer capabilities of the anodic microbial community [5].

To date, most MDCs rely on a limited repertoire of well-characterized exoelectrogens, notably *Geobacter sulfurreducens* and *Shewanella oneidensis*, which possess well-studied extracellular electron transfer (EET) mechanisms [6]. However, these model strains often exhibit sensitivity to environmental stressors such as high salinity, heavy metals, or complex organics—conditions typical of industrial wastewaters. Therefore, the discovery and application of novel, extremotolerant exoelectrogens are essential for improving MDC reliability and scalability in real-world contexts [7,8].

Industrial anaerobic sludges, particularly those derived from petrochemical and oil refining wastewater treatment plants, represent highly selective environments enriched in metals, hydrocarbons, and redox-active compounds. These niches likely harbor underexplored microbial populations with enhanced iron-reducing capacity and metabolic adaptability [9]. Yet, few studies have systematically isolated and evaluated iron-reducing bacteria (IRB) from such environments for bioelectrochemical applications. The *Advenella* Sp. (family Alcaligenaceae, β-Proteobacteria) includes strains with reported resistance to heavy metals and capacity for aromatic compound degradation, suggesting inherent metabolic versatility for harsh environments [10,11]. However, to our knowledge, no member of this genus has been previously described as electroactive or evaluated in MDC systems. This presents a critical gap in our understanding of the ecological and functional diversity of IRB in bioelectrochemical platforms. Moreover, while many MDC studies focus on synthetic NaCl solutions, few have applied real industrial brine to assess system performance under complex salinity and ion competition [12]. Likewise, differential transport efficiencies of key ions (e.g., Na^+^, Ca^2+^, Cl^−^) across membranes and their impact on conductivity, power output, and desalination rate remain insufficiently characterized. Structural and biochemical changes in bacterial cells—such as those induced by iron stress—are also rarely analyzed in connection with MDC function, despite their potential relevance to electron transfer and membrane interaction [13]. While model exoelectrogens like Geobacter and Shewanella are well studied, industrial extremophiles remain underexplored. We hypothesized that iron-rich refinery sludge harbors novel IRB with potential for MDC application[14–17].

In this study, we isolated and characterized a novel iron-reducing bacterium, *Advenella* (*A*.) *faeciporci* strain ZF1, from activated sludge collected at the Esfahan Oil Refining wastewater treatment facility in Iran. This strain exhibited high Fe(III) tolerance (up to 20 g/L), magnetotactic behavior, and PHB granule formation, suggesting strong stress adaptation. We evaluated its phylogenetic identity, physiological traits, and electrochemical performance in a laboratory-scale three-chamber MDC, using both synthetic and real refinery brine as feedwater. Ion removal efficiency, power density, coulombic efficiency, and electrical conductivity were monitored over time. Fourier-transform infrared (FTIR) spectroscopy was used to assess biochemical changes under iron exposure. This work represents the first application of *A. faeciporci* in microbial desalination and provides insight into its potential role as an extremotolerant exoelectrogen for integrated water and wastewater treatment.

## 2. Materials and Methods

### 2.1. Sampling and Isolation of Iron-Reducing Bacteria

Anaerobic sludge samples were collected from the primary anaerobic digestion unit of the Esfahan Oil Refining Company (Esfahan, Iran), an environment characterized by high concentrations of hydrocarbons, sulfides, and heavy metals, particularly iron compounds. Samples were collected in sterile 500 mL glass bottles, purged with N_2_ gas to maintain anoxic conditions, sealed with butyl rubber stoppers, and stored at 4 °C prior to processing. All subsequent enrichment and isolation procedures were performed inside an anaerobic glove box (Coy Laboratory Products, USA) under a N_2_\:CO_2_ (80:20, v/v) atmosphere.

For selective enrichment of iron-reducing bacteria (IRB), 5 mL of sludge was inoculated into 100 mL of modified DSMZ 579 medium, prepared anaerobically and dispensed into 120 mL serum bottles sealed with butyl stoppers. The medium composition (per liter) was as follows: 1.5 g NH_4_Cl, 0.6 g NaH_2_PO_4_, 0.1 g KCl, 2.5 g sodium acetate (as a carbon source), 2.5 g NaHCO_3_, 13.7 g Fe(III) citrate (for initial enrichment) or 5–20 g/L FeCl_3_ (for subsequent trials to assess tolerance), 10 mL trace element solution, 10 mL vitamin solution, and 0.25 mL Na_2_WO_4_·2H_2_O (0.1% w/v). The pH was adjusted to 6.8–7.0 using sterile 1 M NaOH. Cultures were incubated at 28 °C for 7–10 days. Iron reduction was monitored via colorimetric quantification of Fe(II) using the o-phenanthroline assay. Briefly, 1 mL of culture was centrifuged (10,000 × g, 5 min), and 100 μL of the supernatant was mixed with 400 μL of 1 g/L o-phenanthroline solution in 10 mM acetate buffer (pH 4.5). Absorbance was read at 512 nm using a UV–Vis spectrophotometer (Shimadzu UV-1800, Japan). Colonies with the highest Fe(III) reduction activity were streaked onto Fe(III) citrate agar plates under anaerobic conditions and further purified by repeated sub-culturing.

### 2.2. Identification of Isolated Strain

The isolate with the highest iron-reducing capacity, designated ZF1, was subjected to morphological, biochemical, and molecular identification. Cell morphology and Gram reaction were determined via standard Gram staining protocols and observed under a light microscope (1000x magnification, Zeiss Axioscope). For the detection of intracellular PHB granule, ZF1 cultures grown under FeCl_3_ stress (20 g/L) were stained with Sudan Black B and counterstained with safranin [14].

Genomic DNA was extracted from overnight-grown cultures (28 C, NA broth) using the boiling lysis method. The 16S rRNA gene was amplified using universal primers 27F (5’-AGAGTTTGATCCTGGCTCAG-3’) and 1492R (5’-TACCTTGTTACGACTT-3’)]. The 50 L PCR reaction mixture contained: 1x Taq buffer, 1.5 mM MgCl_2_, 0.2 mM dNTPs, 10 pmol of each primer, 1.25 U Taq DNA polymerase (Sinaclon, Iran), and approximately 50 ng of template DNA. The thermal cycling conditions were: initial denaturation at 94 °C for 3 min; 35 cycles of denaturation at 94 °C for 45 s, annealing at 55 °C for 45 s, and extension at 72 °C for 90 s; followed by a final extension at 72 °C for 10 min. The resulting ~1.4 kb amplicons were purified and sequenced (Macrogen, South Korea), then analyzed via the EzBioCloud database and NCBI BLAST. Phylogenetic analysis was conducted using MEGA 6.0 software with neighbor-joining algorithm and 1,000 bootstrap replicates. The sequence was submitted to GenBank under accession number KX639817 [15].

### 2.3. FTIR Spectroscopy

To investigate biochemical responses under iron stress, strain ZF1 was cultivated separately on nutrient agar (NA) and modified Geobacter agar containing 20 g/L FeCl to induce stress. Cultures were incubated at 28 °C for 48 h. After incubation, bacterial biomass was scraped from the agar surface, suspended in deionized water, and centrifuged (10,000 × g, 5 min). The cell pellet was washed twice with deionized water to remove residual salts and media components, then lyophilized. Approximately 2 mg of lyophilized biomass was mixed with 200 mg of spectroscopic-grade KBr and pressed into a transparent pellet. FTIR spectra were acquired using a PerkinElmer Spectrum Two FTIR spectrometer over a range of 4000–400 cm^−1^ at a resolution of 4 cm^−1^, with 32 scans averaged per spectrum. Spectra were baseline-corrected and normalized using OPUS software. Peak assignments were made by referencing established literature on microbial FTIR spectroscopy [16–19].

### 2.4. Microbial desalination Cell (MDC) Setup and Operation

#### 2.4.1. MDC bioreactor construction

**Figure 1.**
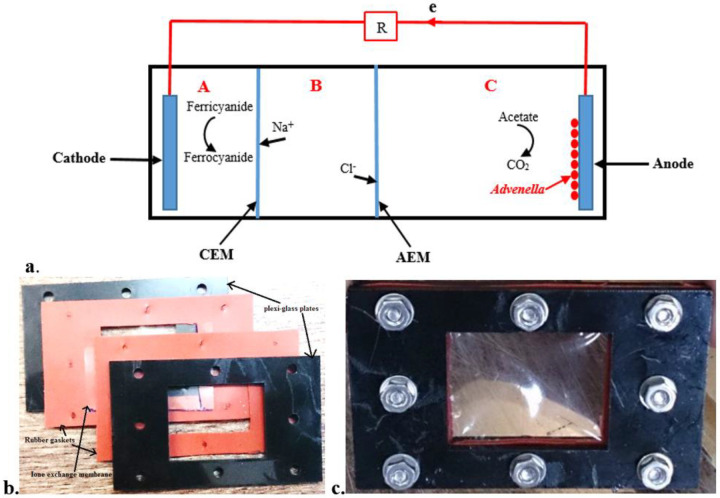
Schematic of the three-chamber MDC. The catholite and anolite chambers are separated by cathion & anion exchange membrane. (a) Overall assembly showing chamber separation by CEM and AEM (plexi-glass plates). (b) Detail of membrane assembly with acrylic plates and rubber gaskets (c) Securing mechanism using 8 stainless steel screws.

A three-chamber MDC was constructed from glass and acrylic components (Fig. 1). The main reactor body measured 12.8 cm × 6.5 cm × 4 cm. The anode and cathode chambers were separated from the central desalination chamber by ion exchange membranes: a Nafion 117 cation exchange membrane (CEM, Sigma-Aldrich, USA) and an A201 anion exchange membrane (AEM, Tokuyama Corporation, Japan). The membranes were positioned 3.2 cm and 6.8 cm from one end of the chamber, respectively, creating three compartments (Fig. 1a) with internal volumes of 80 mL (cathode), 80 mL (desalination), and 160 mL (anode). Each membrane was sandwiched between two acrylic plates and sealed with silicone rubber gaskets, secured by eight stainless steel screws to prevent leakage (Fig. 1b, c).

The projected surface area of each membrane exposed to the solution was 6.9 cm^2^. Carbon cloth (CC-25, 17.5 cm^2^ geometric area) was used as the anode and cathode electrode material. A copper wire connected the anode to a 5 Ω external resistor, which was then connected to the cathode, completing the external circuit. During operation, cations (e.g., Na^+^, Ca^2+^) migrated from the desalination chamber to the cathode chamber through the CEM, while anions (e.g., Cl^−^) migrated to the anode chamber through the AEM.

#### 2.4.2. MDC Medium

The anode and cathode chambers were fed as described by Cao et al [14]. The anolyte solution contained 1.6 g sodium acetate, 3.4 g K_2_HPO_4_. 3H_2_O, 4.4 g KH_2_PO_4_, 1.5 g NH_4_Cl, 0.1 g CaCl_2_. 2H_2_O, 0.1 g MgCl_2_.6H_2_O, 0.1 g KCl and 10 ml of trace mineral solution per liter in deionized water. The catholyte solution contained 16.5 g K_3_Fe(CN)_6_, 8.0 g K_2_HPO_4_. 3H_2_O and 9 g KH_2_PO_4_ per liter deionized water. The desalination chamber was filled by the 80 ml of brackish water of pond 5 of Esfahan Oil Refining company, or NaCl solutions at concentration of 9, 15, 20 or 35 g/L. The anode chamber was inoculated by 10 ml of *Advenella faeciporci* ZF1 strain isolated from Esfahan Oil Refining activated sludge.

#### 2.4.3. Electrochemical characterization

The voltage in the external circuit of the MDC was recorded every 10 minutes 17 using a multimeter (Mod. DEC330FC China). The current density (I) was then calculated according to the Ohm’s law (Eq. 1), 19 and the power density (P) was calculated with (Eq. 2),

Eq. 1 I=V/R

Eq. 2 $${\text{P}=\text{I}}^{2}\cdot \text{R}$$

where I is for current density (mA), V is for the voltage (mV), R is the external resistance (Ω), P is the power density (mW/m2 23) and A stand as the electrode surface area (m^2^) and P is for power density (mW/m^2^). Polarization curves (P versus I) were performed at different moments of the experiment to estimate the enrichment of the exoelectrogenic community and calculate the maximum value of P, which is obtained with the internal resistence (Ω) of the system. The coulombic efficiency, defined as the fraction of electrons recovered as current versus the maximum theoretical recovery from the substrate oxidation, was calculated using data collected after acetate pulses and using (Eq. 3),

Eq. 3 CE=Cp/CTi100

where CE is the coulombic efficiency (%), Cp is the total number of Coulombs estimated by integrating the electric current over time and CTi is the theoretical amount of Coulombs that can be produced from acetate, calculated assuming total removal of the acetate added, similarly as previously described by Luo et al [20,21].

#### 2.4.1. Analysis of Microbial Desalination Cell (MDC) Performance: Electrical Conductivity (EC) and Electrochemical Potential.

The study evaluates the desalination efficiency and electrochemical performance of *Advenella faeciporci* ZF1 in a microbial desalination cell (MDC). Potential (mV), EC (mS/cm) and Current (mA) were recorded every 10 minutes (trends and data based on the described experimental setup) across a 5Ω external resistor using Ohm’s law.

## 3. Results and Discussion

### 3.1. Isolation and Characterization of Strain ZF1

Anaerobic enrichment using refinery sludge supplemented with FeCl_3_ (5–20 g/L) resulted in the successful isolation of multiple iron-reducing bacterial colonies. Among these, strain ZF1 exhibited the most pronounced Fe(III)-reducing capacity, as indicated by rapid development of an orange-red complex with o-phenanthroline in broth assays. The maximum absorbance at 512 nm correlated with Fe(II) accumulation, confirming active dissimilatory iron reduction. Notably, ZF1 was capable of growth under both aerobic and anaerobic conditions; however, quantitative Fe(III) reduction was significantly higher under anaerobic conditions (p < 0.01), consistent with its metabolic role as a facultative iron-reducing bacterium.

**Figure 2 –:**
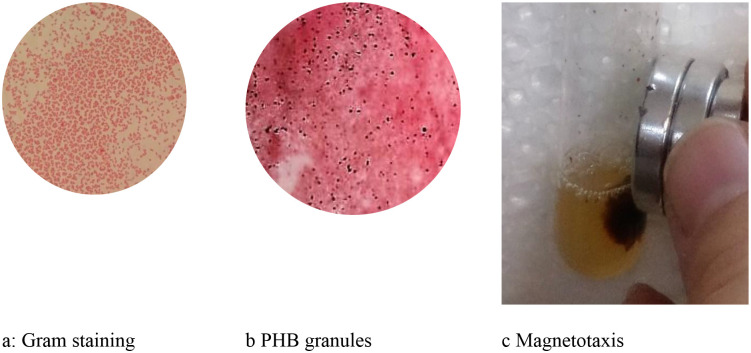
Morphology and Staining of Strain ZF1. 2a: Light microscopy (1000x), Gram stain → Gram-negative cocci. 2b: Sudan Black B stain → PHB granules (black dots), 2c: Magnetotaxis assay → Cell migration in magnetic field

Morphological examination under light microscopy revealed ZF1 to be a Gram-negative, non-sporulating coccus occurring in pairs and short chains (Fig. 2a). Sudan Black B staining demonstrated distinct intracellular accumulation of PHB granules in iron-stressed conditions (Fig. 2b), suggestive of a stress-adaptive carbon storage mechanism. In addition, magnetotactic behavior was observed via directional movement of live cells in response to an external magnetic field (Fig. 2c), implicating the potential presence of magnetosomes or iron-containing intracellular inclusions, which may play a role in environmental sensing or redox processes.

**Figure 3.**
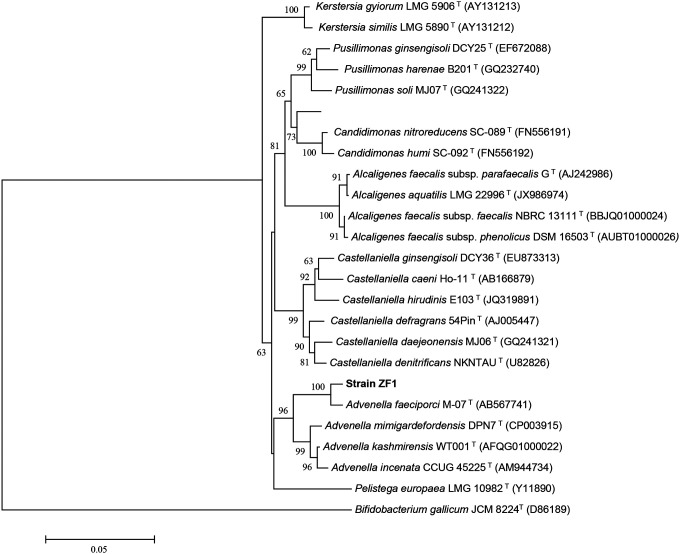
Phylogenetic tree calculated with MEGA 6 from 16S rRNA gene sequences using the neighbour-joining method showing the position of strain ZF1 among the related species. Accession numbers of the sequences are given in parentheses. The sequence of *Bacillus subtilis* DSM10(T) was used as outgroup. Bootstrap values above 50 %, based on 1000 resamplings, are shown at the branching points. Bar, 0.02 substitutions per nucleotide position.

Molecular identification of strain ZF1 was conducted via 16S rRNA gene sequencing. The 1395 bp sequence shared 97.25% identity with *Advenella faeciporci* strain M-07 (GenBank accession AB567741), placing it within the Alcaligenaceae family. Phylogenetic reconstruction using MEGA 6.0 with the neighbor-joining method (1,000 bootstrap replicates) confirmed its taxonomic position within the *Advenella* genus (Fig. 3). The partial 16S sequence was submitted to GenBank under accession number KX639817, supporting the designation of this isolate as *Advenella faeciporci* strain ZF1.

### 3.2. FTIR Spectral Analysis of ZF1 Under Iron Stress

**Figure 4.**
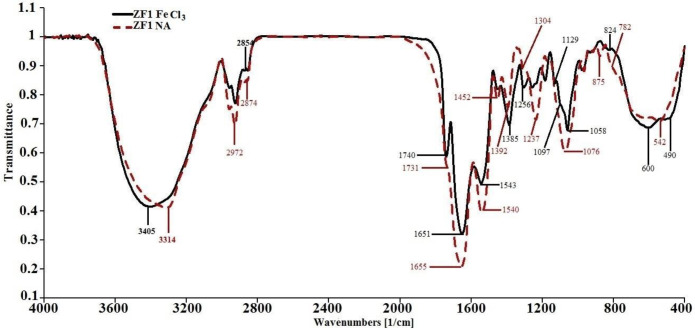
Comparison of FTIR spectra of ZF1 strain in NA and in the presence of FeCl_3_ mixed spectra. Spectral overlay: Control vs. Fe-stressed cells, Annotated peaks: 3405, 1740, 1651, 1543, 1385, 600, 490 cm^−1^, Subfigures: 3a (full spectra), 3b (zoom lipid/protein), 3c (zoom low-frequency) ZF1Fe a 1740 cm^−1^: Strong carbonyl (C=O) stretch, likely from esters, ketones, or carboxylic acid derivatives. b 1385 cm^−1^: Likely C-H bending (e.g., in CH_3_ or CH_2_ groups) or S=O symmetric stretching (sulfonates). c 2959–2854 cm^−1^: Aliphatic C-H stretching (sp^3^ hybridized, typical of alkanes). d 1651 cm^−1^: Possible amide I band (C=O stretch in proteins/polyamides) or conjugated carbonyl. 5. 1543 cm^−1^: Amide II band (N-H bending + C-N stretching, supporting proteinaceous material). 6. 1256–1058 cm^−1^: Likely C-O stretching (esters, ethers) or C-N vibrations. ZF1NB: 1. 1731 cm^−1^: Slightly shifted carbonyl (C=O) stretch compared to ZF1Fe, suggesting a different electronic environment (e.g., ester vs. ketone). 2. 1392 cm^−1^: Similar to ZF1Fe’s 1385 cm^−1^, likely C-H bending or sulfonate group. 3. 2959–2874 cm^−1^: Aliphatic C-H stretching (similar to ZF1Fe). 4. 1655 cm^−1^: Overlaps with amide I/II regions, but weaker intensity may indicate fewer amide bonds. 5. 1304 cm^−1^: Potential C-N stretch (amines, amides) or aromatic vibrations. Comparative Observations 1. Carbonyl Groups: - ZF1Fe (1740 cm^−1^) vs. ZF1NB (1731 cm^−1^): The shift suggests differences in substituents (e.g., ester vs. ketone) or hydrogen bonding. 2. Amide Content: - ZF1Fe shows stronger amide I/II bands (1651, 1543 cm^−1^), indicating potential protein/polyamide content. - ZF1NB lacks distinct amide II peaks, possibly indicating a different backbone structure. 3. Aliphatic vs. Functionalized Regions: - Both samples have strong aliphatic C-H stretches (~2900 cm^−1^), consistent with hydrocarbon chains. - ZF1NB has unique peaks at 1304 cm^−1^ and 1076 cm^−1^, possibly indicating nitro (NO_2_) or phosphonate (P=O) groups.

Fourier Transform Infrared (FTIR) spectroscopy was employed to compare the biochemical composition of ZF1 cells grown under standard conditions (NA) and iron stress (20 g/L FeCl_3_). The spectra revealed significant alterations in functional group profiles, indicative of metabolic and structural adjustments in response to high Fe(III) concentrations (Fig. 4).

Prominent shifts were observed in peaks associated with protein and lipid content. Fe-Stressed cells displayed intensified amide I (1651 cm^−1^) and amide II (1543 cm^−1^) bands, representing protein C=O and N-H vibrations, respectively, suggesting increased protein synthesis or structural remodeling of cell envelope components. These significant shifts in the amide I (1651 cm^−1^) and amide II (1543 cm^−1^) bands indicate enhanced protein synthesis or remodeling, which reflects a response mechanism similar to that seen in heavy metal-resistant strains characterized in Mounaouer et al., where metabolic adjustments in bacteria exposed to pollutants were observed [22]. Stronger ester C=O stretches (1740 cm^−1^) and aliphatic C–H bending (1385 cm^−1^) signals were observed, consistent with elevated PHB content and membrane lipid adaptation. Increase polyhydroxybutyrate content, aligns with findings from other studies on bacterial stress responses involved in biopolymer accumulation under stress conditions [23]. A broad OH stretch band at 3405 cm^−1^ in iron-exposed cells, absent in controls, is attributed to carboxylic acid–iron coordination, implying enhanced metal binding at the cell surface. The OH band related to iron coordination suggests that ZF1 undergoes biochemical changes to stabilize metal ions, similar to mechanisms discussed in studies focusing on bioaccumulation and bioremediation of heavy metals. Additionally, spectral features at 600 cm^−1^ (≡C–H bending) and 490 cm^−1^ (C–Br stretching) suggest stress-related biosynthesis of unusual secondary metabolites or metal-chelating exopolymers (Table 2).

Shifts in FTIR peaks (e.g., carboxylic acid dimer → monomer OH at 3405 cm^−1^) indicate Fe-carboxyl bonding in FeCl_3_-exposed cells. This suggests enhanced metal-binding capacity, potentially improving Fe^3+^ reduction and electron transfer in the MDC. Peaks at 600 cm^−1^ (C≡C-H bend) and 90 cm^−1^ (C-Br stretch) in FeCl_3_-grown cells may reflect stress-response biomolecules aiding ion tolerance. Collectively, these FTIR data support the hypothesis that iron exposure induces both physiological stress responses (e.g., PHB accumulation) and adaptive biochemical modifications that may enhance redox flexibility and cell integrity under MDC-relevant conditions.

### 3.3. Electrochemical Performance of MDC Inoculated with ZF1

The performance of *A. faeciporci* ZF1 in a three-chamber microbial desalination cell (MDC) was evaluated under varying salinity conditions using both synthetic NaCl solutions and real refinery brine.

**Figure 5.**
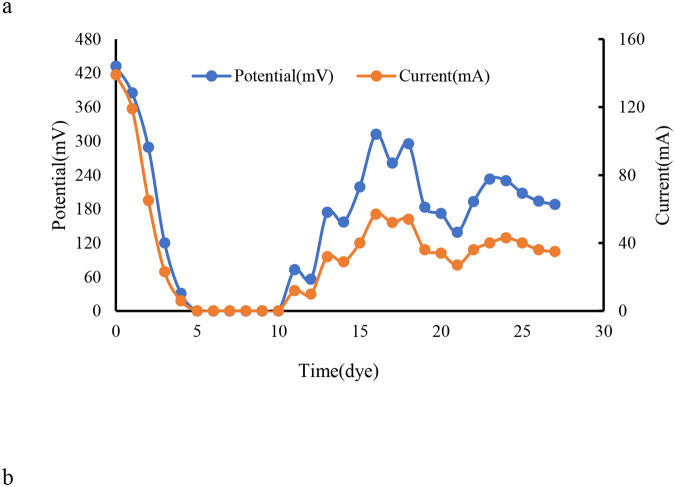

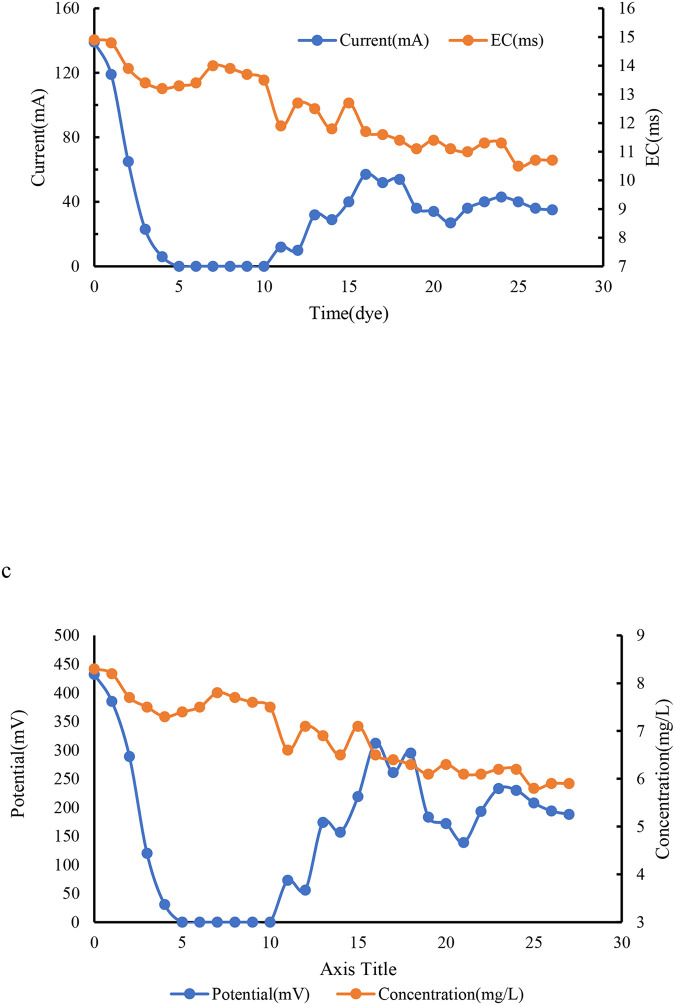
MDC Electrochemical Performance. 5a: Voltage output (mV vs. time) over 15 days, 4b: Power density (mW/m^2^) vs. current density (A/m^2^), 4c: Coulombic efficiency vs. salinity (bar graph with error bars)

Under 9 g/L NaCl salinity, the system generated a maximum voltage of 410 ± 15 mV within the first 48 h (Fig. 5). The corresponding peak power density reached 92 ± 5 mW/m^2^ at an external resistance of 100 Ω, and the calculated current density was 1.80 ± 0.1 A/m^2^. These values are comparable to those reported for well-characterized exoelectrogens in similar setups. Our observation of 410 ± 15 mV at 9 g/L NaCl is comparable to Liu and Logan, who reported robust electricity generation in microbial fuel cells under optimized conditions [24,25]. This suggests that ZF1 is capable of maintaining effective electron transfer even under defined salinity levels.

Coulombic efficiency for this condition was calculated at 61.2%, indicating efficient electron recovery from acetate oxidation. In contrast, increasing NaCl concentration to 35 g/L resulted in reduced electrochemical performance, with peak voltage and power density declining to 275 ± 20 mV and 48 ± 7 mW/m^2^, respectively. CE dropped to 34.5%, likely due to osmotic stress impairing microbial activity, increased internal resistance, and ion accumulation near the membrane surface. Reduced voltage and power density at 35 g/L NaCl highlight osmotic stress impacts, which line up with the concept outlined in Logan [25] regarding the limits of exoelectrogenic bacteria in hyper-saline environments. Increased internal resistance due to membrane fouling resonates with findings in literature about impaired microbial performance under high salinity or competitive ion environments.

These findings highlight the salt-tolerance limits of ZF1 and the performance trade-offs in high-salinity MDC applications. Figure 5 shows initial voltage spikes are expected as the bacteria metabolize acetate, followed by stabilization as substrate availability decreases. Higher NaCl concentrations (e.g., 35 g/L) likely reduced voltage due to increased osmotic stress or membrane fouling. CE measures the fraction of electrons recovered as current versus theoretical maximum from acetate oxidation. A high CE (e.g., >50%) would indicate efficient electron transfer by *A. faeciporci* ZF1, suggesting strong exoelectrogenic activity. Lower CE in high-salinity conditions might reflect diverted metabolic pathways or energy spent on osmoregulation. These curves (power density vs. current density) help determine the internal resistance of the MDC. The maximum power density occurs when external resistance matches internal resistance. Lower internal resistance (e.g., due to efficient ion exchange membranes) correlates with higher power output.

### 3.4. Ion Removal and Conductivity Reduction

**Figure 6 –:**
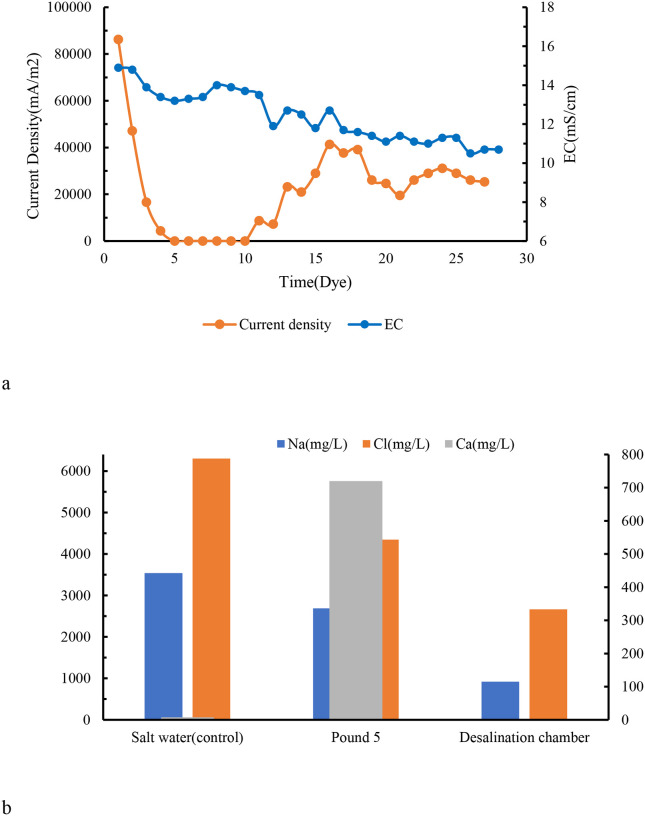
Desalination and Ion Removal. 6a: Conductivity drop (%) over time (line graph), 6b: Ion removal efficiency (Na^+^, Ca^2+^, Cl^−^) for synthetic vs. real brine (bar chart)

Desalination efficiency was assessed by monitoring electrical conductivity (EC) and ion concentrations in the central desalination chamber. EC decreased steadily over time in all tested conditions (Fig. 6a). For 9 g/L NaCl solution, a 58.4 ± 3.2% reduction in EC was achieved within 15 days. In refinery brine, EC decreased by 49.6 ± 4.5%, demonstrating the system’s applicability to complex real-world wastewaters.

As the figure shows, in the desalination of Pond 5, the concentrations of sodium, chloride, and calcium ions decreased by70.63%, 27.47%, and 78.61%, respectively, over 15 days. A similar trend, albeit with a slightly different rate, is observed in the desalination of the 9 g/L brine sample, where sodium, chloride, and calcium levels decreased by 73.95%, 57.74%, and 94.13%, respectively. The greater removal of sodium and calcium cations could be attributed to the higher efficiency of the Nafion cation exchange membrane (CEM) compared to the Tokuyama anion exchange membrane (AEM). Ion chromatography analysis revealed that sodium and calcium ions were effectively removed from both synthetic and real brine, while chloride removal was more limited. In refinery brine, Na^+^, Ca^2+^, and Cl^−^ removal efficiencies were 70.6%, 78.6%, and 27.5%, respectively. In synthetic brine, these values increased to 73.9%, 94.1%, and 57.7% (Fig. 6b). The superior removal of Ca^2+^ and Na^+^ is attributed to the high cation selectivity of the Nafion^®^ CEM, while the lower Cl^−^ removal reflects the comparatively reduced transport efficiency of the AEM and the presence of competing anions in refinery brine. Statistical analysis (p < 0.01) confirmed significant differences in cation versus anion removal, reinforcing the need to optimize membrane selection and system configuration for balanced ion transport. Our findings on ion removal efficacies, particularly the significant reductions in sodium, chloride, and calcium concentrations, can be effectively compared with prior studies. The achieved removal rates (e.g., 70.63% Na^+^, 78.61% Ca^2+^ in real refinery brine) indicate a high effectiveness of the MDC application, which aligns with findings from Mounaouer et al. [26], on the adaptability of bacteria in polluted environments for ion and heavy metal removal. This suggests that ZF1 not only generates electricity but also serves as an effective bioremediator, leveraging its metabolic flexibility.

### 3.5. Integration of Structural and Functional Traits

The combination of physiological flexibility (facultative Fe(III) reduction), biochemical stress responses (PHB synthesis, iron coordination), and adaptive behaviors (magnetotaxis) suggest that *Advenella faeciporci* ZF1 is well-equipped to function under the complex conditions typical of MDC systems. FTIR data provide compelling evidence for iron-induced remodeling of cell wall proteins and lipid architecture, which may facilitate enhanced electron transfer and resilience to high salinity or oxidative stress. Overall, the MDC inoculated with ZF1 demonstrated robust desalination performance and energy generation, particularly under moderate salinity. While high NaCl concentrations impaired performance metrics, ZF1 remained viable and functional, underscoring its potential for treating diverse saline and metal-rich wastewaters. Integration of physiological traits, such as facultative Fe(III) reduction, PHB synthesis, and magnetotaxis, underlines ZF1’s adaptability to complex conditions in MDC systems. This is a common theme in studies like Logan [25], which emphasizes the functional traits of exoelectrogens in bioprocessing systems, indicating the relevance of our findings in the broader context of bioelectrochemical systems. These findings position *A. faeciporci* ZF1 as a promising candidate for next-generation bioelectrochemical systems. Table 3 shows a comparison of MDS.

Future work should explore gene expression patterns during electrochemical operation, long-term biofilm dynamics, and the scalability of ZF1-based systems under continuous-flow conditions. Integration with advanced membrane materials and co-culture strategies could further enhance system efficiency and ion removal selectivity.

## 4. Conclusion

This study reports the successful isolation, identification, and application of *Advenella faeciporci* strain ZF1—a novel iron-reducing bacterium obtained from oil refinery sludge—for microbial desalination. Strain ZF1 demonstrated key physiological traits such as facultative Fe(III) respiration, PHB granule accumulation, and magnetotactic behavior, indicating strong adaptability to metal-rich and anaerobic environments. FTIR spectroscopy revealed profound structural and biochemical responses to iron stress, including enhanced protein and lipid content and metal-binding functionalities, which likely contribute to electrochemical resilience. When integrated into a three-chamber microbial desalination cell, ZF1 facilitated effective voltage generation, with a peak power density of 92 mW/m^2^ and a coulombic efficiency exceeding 60% under moderate salinity. The strain enabled substantial removal of Na^+^, Ca^2+^, and Cl^−^ ions from both synthetic and real refinery brines, reducing overall conductivity by up to 58%. These results underscore ZF1’s capability as a robust bioelectrocatalyst for simultaneous wastewater treatment and desalination.

Taken together, our findings establish *A. faeciporci* ZF1 as a promising microbial platform for energy-efficient desalination in complex, saline, and metal-laden wastewaters. Future research should explore system optimization via membrane innovation, co-culture strategies, and continuous-flow operation to enhance long-term stability and performance in practical environmental settings.

## Figures and Tables

**Table 1:** Comparison of FTIR spectra peaks of ZF1 strain in different media

ZF1: NA Wavenumber(cm^−1^)	Class	Structure	Assignment	ZF1: FeCl_3_ Wavenumber(cm^−1^)
-	Carboxylic Acids	rco- oh	Monomer OH	3405
3314	Carboxylic Acids	c=c-co-oh	Dimer OH	-
2959	Alkanes	-CH_3_	-CH_3_	2959
2927	Alkanes	-CH_2_-	-CH_2_-	2925
2874	Alkanes	-CH_3_	-CH_3_	-
-	Alkanes	-CH_2_-	-CH_2_-	2854
1731	Esters	RCOOR′	C=O stretch	1740^a^
1655	Amides	RCONH_2_	C=O stretch (H-bond)	1651^c^
1540	Amides	RCONHR′	NH out of plane	1543
1452	Aromatics	C-C in ring	Ar C-C stretch	1451
1392	Misc.	S=O sulfate	S=O sulfate ester	1385^b^
1304	Misc.	N-O nitro comp.	N-O symm. stretch	-
-	Alkyl halides	CH_2_X	C-H wag (-CH_2_X)	1259
1237	Alkyl halides	CH_2_X	C-H wag (-CH_2_X)	-
-	Alkyl halides, Amines	CH_2_X, RNH_2_, R_2_NH	C-H wag (-CH_2_X)	1188
1167	Amines, Alkyl halides	RNH2, R_2_NH	C-N stretch	-
-	Amines	RNH2, R_2_NH	C-N stretch	1129
-	Amines	RNH2, R_2_NH	C-N stretch	1097
1076	Amines	RNH2, R_2_NH	C-N stretch	1058
965	Misc.	P-H phosphine	P-H bending	975
875	Aromatics		C-H out of plane	-
-	Aromatics	1,3,5-trisub.	C-H out of plane	824
782	Aromatics	Meta -disub.	C-H out of plane	-
698	Aromatics	monsubst.	C-H out of plane	-
-	Alkynes	RC≡CH	≡C-H bend	600
541	Alkyl halides	R-Br	C-Br stretch	490

**Table 2 –:** FTIR Band Assignments and Intensity Changes

Wavenumber (cm^−1^)	Functional Group	Control Intensity	Fe-Stress Intensity	Assignment
3405	OH stretching	++	++++	Carboxylic acids
1740	C=O ester	++	++++	Lipids / PHB
1651	Amide I	+++	++++	Proteins
1543	Amide II	++	+++	Proteins
1385	ch_2_/ch_3_ bending	++	+++	Lipids
600/490	C-Br, alkynes	+	++	Stress-related compounds

**Table 3 –:** MDC Performance Summary

Parameter	9 g/L NaCl	35 g/L NaCl	Refinery Brine
Max Voltage (mV)	410 ± 15	275 ± 20	295 ± 18
Max Power Density (mW/m^2^)	92 ± 5	48 ± 7	58 ± 6
Coulombic Efficiency (%)	61.2	34.5	43.8
Na^+^ Removal (%)	73.9	64.1	70.6
Ca^2+^ Removal (%)	94.1	83.2	78.6
Cl^−^ Removal (%)	57.7	33.5	27.5
EC Reduction (%)	58.4 ± 3.2	41.9 ± 4.0	49.6 ± 4.5

## Data Availability

The datasets generated and/or analysed during the current study are available in the GenBank repository, accession number KX639817 (https://www.ncbi.nlm.nih.gov/nuccore/KX639817).
